# The *in vivo* genotoxicity of isotretinoin assessed by comet assay

**DOI:** 10.17179/excli2019-2053

**Published:** 2020-02-10

**Authors:** Maede Bordbar, Zahra Zendeh-Boodi, Mostafa Saadat

**Affiliations:** 1 Department of Biology, College of Sciences, Shiraz University, Shiraz 71467-13565, Iran

## ⁯⁯

***Dear Editor,***

Isotretinoin (13-cis-retinoic acid) is a vitamin A derivative, which is used as the most effective medication in the treatment of acne vulgaris. Its anti-acne action exerts through reduction of sebum excretion, inhibition of comedogenesis, and decrease in the number of *Propionibacterium acnes* population (Rigopoulos et al., 2010[6]). Despite its excellent efficacy, the use of isotretinoin for acne due to its deleterious side effects such as oxidative stress, teratogenesis, mucosa and mood disorders remains controversial (Layton, 2009[4]; Erturan et al., 2012[2]). The evidence related to the genotoxicity of isotretinoin are controversial. While inducing apoptosis and necrosis at higher doses in *in vitro* studies, isotretinoin had no genotoxic effect when tested in human lymphocytes (Olive and Banáth, 2006[5]). It has been reported that patients with cystic acne showed elevated level of plasma biomarker of DNA oxidation, after treatment by isotretinoin (Silva et al., 2013[7]). However, the controversial effect of isotretinoin on frequency of micronuclei was reported (Benner et al., 1994). Thus, further elucidation of isotretinoin biological effects may help to reach a conclusion on its use. Therefore, the present study was carried out.

The comet assay is a technique that can measure DNA damage such as single strand breaks, double strand breaks, crosslinks and base damage for individual eukaryotic cells (Georgala et al., 2005[3]). The purpose of this study is to evaluate the extent of DNA damage in leukocytes of patients on isotretinoin treatment. To this aim, a fresh blood sample was obtained from 26 volunteers, including 13 (11 females, 2 males) healthy individuals and 13 (10 females, 3 males) patients with acne on isotretinoin treatment. All of the participants were Persian (Caucasians) Muslims living in Fars province (south west of Iran). Written informed consent was obtained from each participant. This study was approved by the Shiraz University Ethics Committee. 

Then, the Olive & Banáth's comet assay protocol (Georgala et al., 2005[3]) with some modifications was performed on the participant's isolated mononuclear leucocytes. After preparation of the slides, images of 100 randomly chosen cells were captured per sample (Nikon fluorescence microscope) and DNA damage was evaluated by measuring the tail length (TL), tail DNA percent (TD), and tail moment (TM) using TriTek CometScore V 2.0 software. Independent samples *t*-test and Χ^2^ were performed using the SPSS software (SPSS Inc., Chicago, IL, USA) (version 26). Statistical analyses were two-tailed and the level of significance was set at P<0.05. 

The mean ± SE (standard error) of age of the controls and the patients were 23.6 ± 0.65 and 21.7 ± 0.66 years, respectively. There was no significant difference between the study groups for age (t=2.05, df=24, P=0.051) and gender (Χ^2^=0.24, df=1, P=0.619).

The values (mean ± SE) of TL, TD and TM in study groups were summarized in Table 1(Tab. 1). Kolmogorov-Smirnov test indicating that age of participants, TL, TD and TM have normal distribution (For age: Kolmogorov-Smirnov Z-test=0.138, P=0.200; For TL: Kolmogorov-Smirnov Z-test=0.109, P=0.200; For TD: Kolmogorov-Smirnov Z-test=0.170, P=0.052; For TM: Kolmogorov-Smirnov Z-test=0.112, P=0.200). Statistical analysis indicated that no significant difference in any parameters was found between the two groups (Table 1(Tab. 1)). 

The results of the present study did not show the genotoxicity effect of isotretinoin on the patient's leucocytes. Although our data did not support the genotoxicity of isotretinoin, further studies are required to confirm the safety of this drug. 

## Acknowledgements

The authors are indebted to the participants for their close cooperation. This work was supported by the Shiraz University, Iran.

## Conflicts of interest

None. 

## Figures and Tables

**Table 1:**
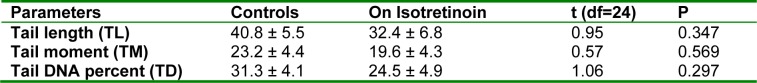
Tail length, tail DNA percent, and tail moment in participants receiving or not receiving isotretinoin
